# Extensive facial necrosis after infiltration of polymethylmethacrylate

**DOI:** 10.1016/S1808-8694(15)31184-8

**Published:** 2015-10-19

**Authors:** Anderson Castelo Branco de Castro, Marcus Vinicius Martins Collares, Ciro Paz Portinho, Paulo Cesar Dias, Rinaldo di Angeli Pinto

**Affiliations:** 1 MD. ENT. Fellow - Cranio-Maxillo-Facial Surgery - H. Clínicas de Porto Alegre - RS; 2 MD. Plastic Surgeon and Cranio-Maxillo-Facial Surgeon. MSc. And PhD in Plastic Surgery; 3 MD. Plastic Surgeon. M.Sc. in Plastic Surgery - UFRGS; 4 MD. ENT. M.Sc. in Otorhinolaryngology. Fellow - Cranio-Maxillo-Facial Surgery - H. Clínicas de Porto Alegre - RS; 5 M.D. Plastic Surgeon. Head of the Plastic Surgery Department - University Hospital - Porto Alegre - RS; Professor of the Graduate Course in Cranio-Maxillo-Facial Surgery - University Hospital - Porto Alegre - RS

**Keywords:** face, necrosis, polymethylmethacrylate

## INTRODUCTION

Polymethylmetacrylate (PMMA) is a synthetic polymer that is being used in large scale in cosmetic surgery to fill soft tissue. In its injectable form, it is made up of microspheres spread within a colloid medium of carboxymethylcellulose. It is used to soften wrinkles, increase volume and enhance facial contour, among other surgical applications.

We report a case of a 77-year-old lady who had a necrosis in the right side of her face after PMMA injection in the nasogenian groove.

## CASE PRESENTATION

ZMS, 77 years of age, came to the emergency room of a private hospital in Porto Alegre - RS, on March 26, 2005, with facial pain, edema and cyanosis in her right hemi face. Approximately 24 hours before she had been submitted to a cosmetic treatment on her face, a bioplasty with PMMA, performed by an experienced plastic surgeon.

After being admitted, the patient was initially under the care of the vascular surgery team, they suspected of a facial artery embolism caused by PMMA and started her on full heparinization and analgesia.

During hospital stay, the extensive cyanosis area on her right hemi face reduced a little and then there was tissue necrosis in the areas nurtured by the following arteries: right inferior and superior labial coronal arteries, right angular artery.

On March 29, 2005, the Cranio-Maxillo-Facial Surgery team of the hospital was contacted. After assessment, they debrided the necrotic areas on her right hemi face on March 30 of 2005. They debrided the nasal wing on the right side, and the right side of the upper and lower lips. The patient remained in the hospital for three more weeks undergoing daily dressings on the debrided area. During this period, she still suffered one more debridement in the surgical center because of the extension of the necrosis on her right nasal wing. During the healing period, she complained of much pain in her right side mandible because her right chin nerve was exposed in the open tissue.

Our team is currently following her up.

## DISCUSSION

Bioplasty started in the 90's in the USA, with the American surgeon Dr. Robert Ersek. He described the use of bioplastique, a biocompatible polymer, and he also developed non-traumatic canulas for its application. Ersek also described the ideal material characteristics to be used in bioplasty: bio-inertia, permanent, particle size large enough to prevent tissue migration, particle size small enough to pass through the microcanula, clear color, moldable for implant and stable afterwards.^1^

**Figure:**
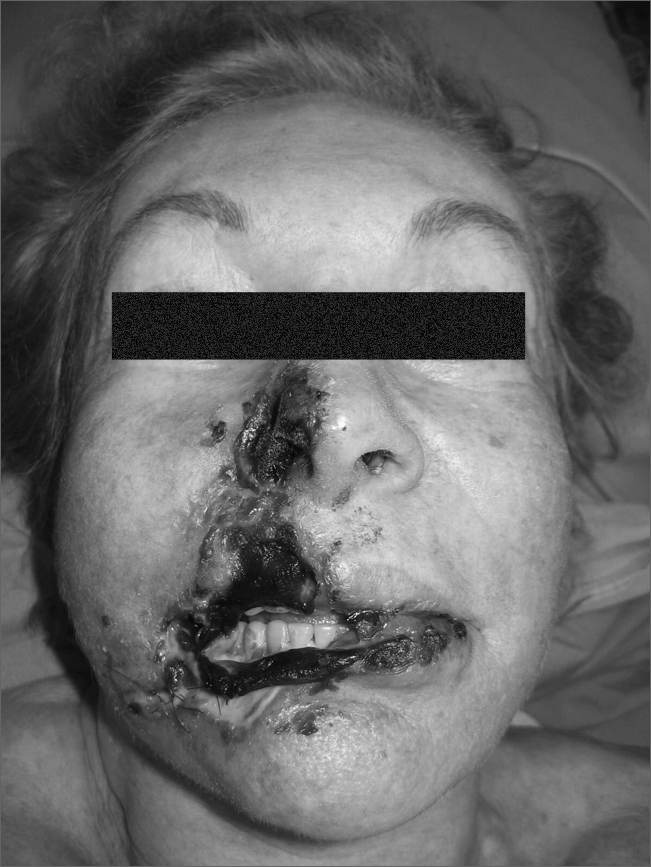
Complication after PMMA injection - Notice the extensive postoperative necrotic area on the right hemi face (nasal wing, upper and lower lips, and part of the buccinators region).

PMMA is one of the most used substances in bioplasty. It is made up of microspheres of about 40 micra suspended in a colloid medium of carboxymethylcellulose, non-absorbable and free from protein material. Its use became popular for facial rejuvenation, since it smoothens wrinkles, increases tissue volume and enhances facial contour.^2^

The most frequent complications associated with the use of PMMA stem from the polymer resorption when the microspheres are phagocyted, and also when there are local adverse reactions and granulomas are formed.^3^, ^4^, ^5^

Silva and Curi described a case of blindness after glabellar injection of PMMA. It is a rare complication, however, extremely severe if we consider that bioplasty is seen by many patients and surgeons as a practically innocuous procedure and risk-free. These authors concluded that the PMMA microspheres were injected very near branches of the ophthalmic artery, which caused embolism in the vassel.^2^ We also know of complications also associated with the use of an autologous graft when the injection is carried out in the glabellar region.^6^

In this report, the complication mechanism was similar. PMMA was injected in the nasogenian groove, or directly into the right facial artery, or very near it. There was arterial embolism: right angular artery, right superior and inferior labial coronal arteries.

## FINAL REMARKS

This is the first case described of such a severe complication on the face, associated with the use of microspheres from PMMA. This report also shows the risk of injections in the nasogenian region, especially with alloplastic material used in bioplasty.
